# Candidiasis and other oral mucosal lesions during and after interferon therapy for HCV-related chronic liver diseases

**DOI:** 10.1186/1471-230X-12-155

**Published:** 2012-11-02

**Authors:** Yumiko Nagao, Kouji Hashimoto, Michio Sata

**Affiliations:** 1 Department of Digestive Disease Information & Research, Kurume University School of Medicine, 67 Asahi-machi, Kurume, Fukuoka, 830-0011, Japan; 2 Department of Laboratory Medicine, Kurume University Hospital, Kurume, Fukuoka, 830-0011, Japan; 3 Division of Gastroenterology, Department of Medicine, Kurume University School of Medicine, Kurume, Fukuoka, 830-0011, Japan

## Abstract

**Background:**

Oral lichen planus (OLP) is seen frequently in patients with hepatitis C virus (HCV) infection. The aim of this study was to evaluate the occurrence of oral candidiasis, other mucosal lesions, and xerostomia during interferon (IFN) therapy for HCV infection.

**Methods:**

Of 124 patients with HCV-infected liver diseases treated with IFN therapy in our hospital, 14 (mean age 56.00 ± 12.94 years) who attended to receive administration of IFN once a week were identified and examined for *Candida* infection and other oral lesions and for the measurement of salivary flow. Serological assays also were carried out.

**Results:**

Cultures of *Candida* from the tongue surfaces were positive in 7 (50.0%) of the 14 patients with HCV infection at least once during IFN therapy. *C*. *albicans* was the most common species isolated. The incidence of *Candida* during treatment with IFN did not increase above that before treatment. Additional oral mucosal lesions were observed in 50.0% (7/14) of patients: OLP in three (21.4%), angular cheilitis in three (21.4%) and recurrent aphthous stomatitis in one (7.1%). OLP occurred in one patient before treatment with IFN, in one during treatment and in one at the end of treatment. 85.7% of the oral lesions were treated with topical steroids. We compared the characteristics of the 7 patients in whom *Candida* was detected at least once during IFN therapy (group 1) and the 7 patients in whom *Candida* was not detected during IFN therapy (group 2). The prevalence of oral mucosal lesions (P=0.0075) and incidence of external use of steroids (P=0.0308) in group 1 were significantly higher than in group 2. The average body weight of group 1 decreased significantly compared to group 2 (P=0.0088). Salivary flow decreased in all subjects throughout the course of IFN treatment and returned at 6^th^ months after the end of treatment. In group 1, the level of albumin at the beginning of the 6^th^ month of IFN administration was lower than in group 2 (P=0.0550). According to multivariate analysis, one factor, the presence of oral mucosal lesions, was associated with the detection of *Candida*. The adjusted odds ratio for the factor was 36.00 (95% confidence interval 2.68-1485.94).

**Conclusion:**

We should pay more attention to oral candidiasis as well as other oral mucosal lesions, in patients with weight loss during IFN treatment.

## Background

Hepatitis C is a major global public health problem and hepatitis C virus (HCV) infection is one of the main causes of cirrhosis and hepatocellular carcinoma (HCC). Interferon (IFN) therapy reduces the incidence of hepatocarcinogenesis in patients with hepatitis C and improves the long-term prognosis [1-4]. The standard therapy is a combination of pegylated interferon (Peg-IFN) and ribavirin (RBV). In addition, Peg-IFN, RBV, and telaprevir triple therapy is a new strategy expected to eradicate the HCV even in patients infected with difficult-to-treat genotype 1 strains, although adverse effects, such as anemia and rash, are frequent [5,6].

Extrahepatic manifestations of HCV infection are numerous [7-10]. The most common diseases associated with HCV are hematologic, dermatologic, renal, and rheumatologic diseases.

Lichen planus (LP) is a chronic and mucocutaneous disease that can affect the oral mucosa, skin, genital mucosa, scalp and nails. A significant association between LP and chronic HCV infection has been reported in Japan, Italy, and Spain [11-16]. Previously, we reported that HCV was associated closely with extrahepatic manifestations such as oral LP (OLP) [12] and reported the examination of hepatitis C patients for oral lesions before, during and after IFN treatment [17,18]; OLP was seen in 11.7 (11/94)-16.7% (4/24). OLP can occur, become exacerbated and persist during IFN treatment for hepatitis C, even when serum HCV RNA becomes negative. Our study suggested that OLP pathogenesis in hepatitis C may be attributed to host factors induced by HCV infection, rather than direct pathological effects of the virus [17].

*Candida* generally causes no problems in healthy people. *Candida* is commonly cultured from oral mucosal lesions. Overgrowth of *Candida*, however, can lead to local discomfort, an altered taste sensation, dysphagia from esophageal overgrowth resulting in poor nutrition, slow recovery, and prolonged hospital stays [19]. Risk factors associated with symptomatic candidiasis include: local or systemic immunosuppression, haematological disorders, broad-spectrum antibiotic use, inhaled or systemic steroids, xerostomia, diabetes, wearing dentures, obturators and orthodontic appliances, and smoking [20-23]. *Candida* is has been reported to be found in 37.0% to 76.7% of OLP cases [24,25]. The treatment of OLP with steroids is known to lead to secondary yeast infection, which may complicate the treatment of OLP. *C*. *albicans*, *C*. *glabrata* and *C*. *tropicalis* have been isolated from OLP lesions treated with topical steroids [25].

Xerostomia is known as one of the adverse events observed during IFN therapy [26,27]. Aghemo et al. reported that dry mouth occurring during IFN therapy results from a reversible inhibition of salivary gland function and that RBV causes salivary gland hypofunction [27]. We also reported the salivary flow rate during IFN therapy [18,28]. In a Japanese Phase III trial of Peg-IFN alfa-2a and RBV involving 199 patients with chronic hepatitis C, including 99 patients with IFN treatment-naive genotype 1 and 100 patients with patients whom had not had a SVR after IFN therapy, 6.5% of all patients developed dry mouth. On the other hand, in a Japanese Phase III trial of Peg-IFN alfa-2b and RBV involving 332 chronic hepatitis C patients, including 269 patients for 48 weeks treatment duration with genotype 1b and high virus load, and 63 patients for 24 weeks treatment duration with others, 15.6% of all patients developed dry mouth.

There have been few reports on oral *Candida* before, during and after IFN treatment for chronic hepatitis C patients. The aims of this study were to determine the incidence of *Candida* as well as the development of OLP, angular cheilitis, recurrent aphthous stomatitis, and xerostomia, in the course of IFN treatment of patients with chronic hepatitis C.

## Materials and methods

### Patients

A total of 124 consecutive HCV-infected patients for whom IFN therapy was planned had checkups for oral mucosal diseases, based on our hospital’s clinical pathway in the Digestive Diseases Center at Kurume University Hospital Japan from July 30, 2008 to October 28, 2009. In this Digestive Diseases Center, physicians, surgeons, radiologists and an oral surgeon examine each patient in their own specialized area.

Seventeen of 124 patients agreed to the simultaneous consultation of the hepatologist and oral surgeon in our Digestive Diseases Center in order to receive administration of Peg-IFN and an oral examination once per week. The other 107 patients did not consult a doctor every week. Fourteen patients completed therapy. Three patients discontinued IFN treatment because of null-response. Systemic autoimmune disorders such as autoimmune hepatitis, and Sjögren’s syndrome were considered as exclusion criteria for IFN therapy.

The 14 patients, 3 men and 11 women, who completed IFN therapy were examined. They ranged in age from 25 to 70 years, with an average age of 56.00 ± 12.94 years. Eleven patients with HCV genotype 1 received administration of Peg-IFN/RBV for 48-72 weeks. Two patients with HCV genotype 2 received administration of Peg-IFN/RBV for 24 weeks, and one patient with liver cirrhosis (HCV genotype 2) received administration of IFN beta for 24 weeks.

### Methods

#### Examination of oral mucosal disease

We questioned the subjects about their frequency of tooth-brushing. We used the headband fiber (50-100-10, Daiichi Medical Co., Ltd.) with a brightness of 34,000 luces for mucosal examination. Oral biopsy was performed on some patients. The diagnosis of OLP was made on the basis of clinical and/or histopathological features. All patients were examined before IFN therapy, at the beginning of the 2^nd^ week of IFN administration, at the beginning of the 3^rd^ month of IFN administration, at the beginning of the 6^th^ month of IFN administration, at the end of IFN therapy, and at 6^th^ months after the end of treatment. The patients were weighed at each hospital visit. When a patient complained of haphalgesia and a burning sensation from oral musocal lesions, we prescribed external steroids, such as 0.1% Dexamethasone (Dexaltin® Nippon Kayaku Co., Ltd, Tokyo, Japan), Triamcinolone acetonide (Aphthaseal® Taisho Toyama Pharmaceutical Co., Ltd, Tokyo, Japan), and Beclometasone dipropionate (Salcoat® Teijin Pharma Co., Ltd, Tokyo, Japan).

#### Salivary flow

Salivary flow was measured in all patients. We used a simple and low-cost test for detection of xerostomia and this required chewing on a piece of gauze for 2 min. A salivary flow rate of below 2 g/2 min was judged as decreased salivary secretion. All patients were examined before IFN therapy, at the beginning of the 2^nd^ week of IFN administration, at the beginning of the 3^rd^ month of IFN administration, at the beginning of the 6^th^ month of IFN administration, at the end of IFN therapy, and at 6^th^ months after the end of treatment.

#### Candida isolation

We examined *Candida* species on the surface of the tongue in 14 patients with chronic liver diseases before, during, and after IFN therapy. The surface of the tongue was rubbed ten times with cotton swabs and these were placed on CHROMagar *Candida* slants. CHROMagar *Candida* medium (CHROMagar, Paris, France) was prepared according to the manufacturer’s instructions. The swab was inoculated into CHROMagar *Candida* medium by rotating the swab head on the surface of the medium. The plates were incubated at 35^o^C for 48 h. Colony morphology and colour description were assigned in a standard manner by a single investigator.

When a patient diagnosed with candidiasis complained of a burning sensation in the oral cavity, we administered an antifungal agent, such as Miconazole (Florid® Mochida Pharmaceutical Co., Ltd, Tokyo, Japan) and Itraconazole (Itrizole® Janssen Pharmaceutical K.K., Tokyo, Japan).

#### Serological assays

At each visit to our hospital for administration of IFN, the patients were tested for platelets (PLT), white blood cell (WBC), and hemoglobin (Hb), and for the following liver function tests: serum alanine aminotransferase (ALT), aspartate aminotransferase (AST), lactate dehydrogenase (LDH), total bilirubin (T.Bil) and albumin (Alb). The presence of anti-SS-A and anti-SS-B antibodies in all patients was tested by using an enzyme-linked immunosorbent assay (ELISA). The normal levels of anti-SS-A (SS-A/Ro(E)[S]; TFB, Ink, Japan) and anti-SS-B (SS-B/La(E)[S]; TFB, Ink, Japan) are less than 10 U/mL, respectively.

#### Evaluation of liver diseases

HCV RNA was analyzed in serum by quantitative PCR assay (COBAS AMPLICOR HCV MONITOR v 2.0 Test, COBAS AmpliPrep/COBAS Taq-Man HCV Test, Roche Molecular Systems, New Jersey, US) [29,30]. HCV genotype was determined by polymerase chain reaction assay, using a mixture of primers for the subtype, as reported previously [31]. Ultrasonographic examination was performed on all patients in order to investigate the shape of the liver and lesions occupying the liver. Computed tomography and liver biopsy were performed on some patients. We used other possible predictors of progression of liver cirrhosis, including serum Alb, T.Bil, prothrombin time, and PLT.

### Ethical considerations

This investigation was undertaken with the understanding and consent of each participating subject and has been conducted in full accordance with ethical principles of the World Medical Association Declaration of Helsinki. The Ethical Committee of Kurume University concluded that this study was not included in an ethic guideline because this study is a medical practice aimed for diagnosis and treatment.

### Statistical analysis

All data are expressed as mean ± standard error. Differences between the two groups were analyzed using the Mann-Whitney U test, Wilcoxon’s test, and the Fisher’s exact test. Differences were judged significant for p <0.05 (two-tailed). Adjusted odds ratios were calculated using logistic regression analysis. All statistical analyses were conducted using JMP Version 6 (SAS Institute, Cary, NC, USA). The level of statistical significance was defined as 0.05.

## Results

### Incidence of positive *Candida* cultures and development of oral mucosal lesions

Cultures of *Candida* from the tongue surfaces were positive in seven (50.0%) of the 14 patients with HCV infection at least once during IFN therapy. Table 1 shows the association of the incidence of positive *Candida* cultures and IFN therapy. The incidence of positive *Candida* cultures was 42.86% (6 cases, *C*. *albicans*) before IFN therapy, 7.14% (1 case, *C*. *albicans*) at the beginning of the 2^nd^ week of IFN administration, 35.71% (4 cases, *C*. *albicans*; 1 case, *C*. *albicans* and *C*. *parapsilosis*) at the beginning of the 3^rd^ month of IFN administration, 42.86% (6 cases, *C*. *albicans*) at the beginning of the 6^th^ month of IFN administration, 35.71% (4 cases, *C*. *albicans*; 1 case, *C*. *lusitaniae*) at the end of IFN therapy and 21.43% (2 cases, *C*. *albicans*; 1 case, *C*. *albicans* and *C*. *tropicalis*) at 6^th^ months after the end of treatment. *C*. *albicans* was the most common species isolated from tongue surfaces. The incidence of *Candida* during treatment with IFN did not increase above that before treatment.

**Table 1:** Characteristics of the subjects

**No.**	**Age**	**Sex**	**Liver Diagnosis**	**HCV genotype**	**HCV RNA level**	**IFN**	***Candida***													**Oral mucosal lesions**	**Study group 1/2**
							**Before IFN**	**colony (n)**	**2 W after IFN**	**colony (n)**	**3 M after IFN**	**colony (n)**	**6 M after IFN**	**colony (n)**	**end of treatment**	**colony (n)**	**6 M after end of treatment**	**colony (n)**	**Detection during IFN therapy (at least once)**		
1	70	F	CH-C	1b	High	Peg-IFN/RBV	−		−		−		−		−		−		−	−	group 2
2	52	M	CH-C	1b	High	Peg-IFN/RBV	+		+		+		+		+		+		+	+	group 1
							*C*. *albicans*	3	*C*. *albicans*	1	*C*. *albicans*	1	*C*. *albicans*	1	*C*. *albicans*	1	*C*. *albicans*	2			
																	*C*. *tropicalis*	1			
							OLP		OLP		OLP		OLP		OLP		OLP				
3	33	F	CH-C	1b	High	Peg-IFN/RBV	+		−		−		−		−		−		−	−	group 2
							*C*. *albicans*	2													
4	53	F	LC-C	ND (serogroup 2)	Low	IFN beta	−		−		−		−		−		−		−	−	group 2
5	70	F	CH-C	2a	High	Peg-IFN/RBV	+		−		+		+		+		−		+	+	group 1
							*C*. *albicans*	1			*C*. *albicans*	1	*C*. *albicans*	2	*C*. *albicans*	2					
											*C*. *parapsilosis*	1									
													angular cheilitis								
6	60	F	CH-C	2a	High	Peg-IFN/RBV	−		−		−		−		−		−		−	−	group 2
7	57	F	CH-C	1b	High	Peg-IFN/RBV	+		−		+		+		+		+		+	+	group 1
							*C*. *albicans*	2			*C*. *albicans*	1	*C*. *albicans*	1	*C*. *albicans*	1	*C*. *albicans*	1			
											recurrent aphthous stomatitis		recurrent aphthous stomatitis		recurrent aphthous stomatitis						
8	58	F	CH-C	1b	High	Peg-IFN/RBV	−		−		−		+		−		−		+	−	group 1
													*C*. *albicans*	1							
9	25	F	CH-C	1b	High	Peg-IFN/RBV	−		−		−		−		−		−		−	−	group 2
10	54	F	CH-C	1b	High	Peg-IFN/RBV	−		−		−		−		−		−		−	+	group 2
															OLP		OLP				
11	61	F	CH-C	1b	High	Peg-IFN/RBV	+		−		+		+		−		−		+	+	group 1
							*C*. *albicans*	1			*C*. *albicans*	1	*C*. *albicans*	1							
											angular cheilitis										
12	69	F	CH-C	1b	High	Peg-IFN/RBV	+		−		+		+		+		+		+	+	group 1
							*C*. *albicans*	1			*C*. *albicans*	1	*C*. *albicans*	1	*C*. *albicans*	1	*C*. *albicans*	1			
													angular cheilitis								
13	60	M	CH-C	1b	High	Peg-IFN/RBV	−		−		−		−		−		−		−	−	group 2
14	62	M	CH-C	1b	Low	Peg-IFN/RBV	−		−		−		−		+		−		+	+	group 1
															*C*. *lusitaniae*	1					
											OLP		OLP		OLP		OLP				
Incidence of Candida or mucosal lesions (%)							42.86%		7.14%		35.71%		42.86%		35.71%		21.43%		50.00%	50.00%	
Total colonies (n)								10		1		6		7		6		5			
*C*. *albicans* (the number of patients)							6		1		5		6		4		3				
*C*. *parapsilosis* (the number of patients)							0		0		1		0		0		0				
*C*. *lusitaniae* (the number of patients)							0		0		0		0		1		0				
*C*. *tropicalis* (the number of patients)							0		0		0		0		0		1				

HCV, hepatitis C virus; CH-C, chronic hepatitis C; LC-C, liver cirrhosis type C; OLP, oral lichen planus; C., *Candida*; IFN, interferon; Peg-IFN, pegylated interferon; RBV, ribavirin; M, male; F, female; ND, not detected; −, negative; +, positive.

Oral mucosal lesions were observed in 50.00% (7/14) of patients: three with OLP (21.43%, 2 men and 1 woman), three with angular cheilitis (21.43%, 3 women) and one with recurrent aphthous stomatitis (7.14%, 1 woman). OLP occurred in one patient before treatment with IFN, in one during treatment and in one at the end of treatment (Table 1). Patient no. 2 (a 52 year old male) had erosive OLP before IFN therapy; his lesions became exacerbated during IFN therapy but he was able to complete the therapy because of improvement with steroid application.

We administered antifungal agents to two patients (nos. 2 and 5 in Table 1) with candidiasis who complained of burning pain and prescribed external steroids for six patients (nos. 2, 5, 7, 10, 11, and 12 in Table 1) with oral mucosal lesions.

### Comparison between *Candida* positive and negative groups

We compared the characteristics of the 7 patients (group 1) in whom *Candida* was detected at least once during IFN therapy and the 7 patients (group 2) in whom *Candida* was not detected during treatment (Table 2). The prevalence of oral mucosal lesions and incidence of external use of steroids were 85.71% (6/7) and 71.43% (5/7), respectively, in group 1 and 14.29% (1/7) and 14.29% (1/7), respectively, in group 2. The prevalence of oral mucosal lesions (P=0.0075) and incidence of external use of steroids (P=0.0308) was significantly higher in group 1 than group 2. The diagnosis of oral mucosal disease in group 1 included: OLP (n=2), angular cheilitis (n=3), and recurrent aphthous stomatitis (n=1). That in group 2 was OLP (n=1).

**Table 2:** **Comparison between *Candida *positive and negative groups**

		**Total**	**Goup 1**	**Group 2**	**P value**
Subjects (n)		14	7	7	
Sex (M/F)		3/11	2/5	1/6	NS
Age (mean ± SD)		56.00 ± 12.94	61.30 ± 6.47	50.71 ± 16.00	NS
HCV genotype	1b	11 (78.57%)	6 (85.71%)	5 (71.43%)	NS
	2a	2 (14.29%)	1 (14.29%)	1 (14.29%)	NS
	ND (serotype 2)	1 (7.14%)	0 (0%)	1 (14.29%)	NS
HCV RNA level	High	12 (85.71%)	6 (85.71%)	6 (85.71%)	NS
	Low	2 (14.29%)	1 (14.29%)	1 (14.29%)	NS
Weight loss during IFN therapy		5.69 ± 4.57	8.71 ± 2.81	2.66 ± 4.00	**0.0088**
Neutrophil count (μ/L)	Before IFN	2546.11 ± 793.47	2481.34 ± 599.67	2610.89 ± 997.36	NS
	2 weeks after IFN	1229.11 ± 590.25	1260.51 ± 473.58	1197.70 ± 726.82	NS
	3 months after IFN	1343.05 ± 584.48	1636.51 ± 670.69	1049.59 ± 298.98	NS
	6 months after IFN	1319.11 ± 301.15	1442.69 ± 194.15	1195.53 ± 350.95	NS
	End of treatment	1360.21 ± 547.74	1420.54 ± 401.41	1299.87 ± 693.13	NS
	6 months after treatment	2349.21 ± 695.36	2578.63 ± 716.47	2119.80 ± 641.48	NS
PLT (x10-4/μL)	Before IFN	17.58 ± 7.06	17.54 ± 3.32	17.61 ± 9.84	NS
	2 weeks after IFN	14.04 ± 7.28	14.37 ±4.70	13.71 ± 9.61	NS
	3 months after IFN	13.69 ± 6.08	14.27 ± 5.36	13.11 ± 7.12	NS
	6 months after IFN	13.20 ± 5.31	14.21 ± 4.72	12.19 ± 6.03	NS
	End of treatment	14.45 ± 5.05	14.50 ± 4.59	14.40 ± 5.84	NS
	6 months after treatment	16.86 ± 6.03	18.14 ± 4.29	15.59 ± 7.52	NS
Hb (g/dL)	Before IFN	13.97 ±1.08	13.91 ±1.26	14.03 ± 0.97	NS
	2 weeks after IFN	13.04 ±1.06	13.33± 1.34	12.76 ± 0.67	NS
	3 months after IFN	10.94 ±1.55	10.40 ±1.57	11.47 ±1.45	NS
	6 months after IFN	10.61 ±1.73	9.81 ± 1.45	11.41 ± 1.70	NS
	End of treatment	10.99 ±1.74	10.23 ± 1.89	11.74 ± 1.27	NS
	6 months after treatment	13.29 ±1.48	12.99 ± 2.05	13.59 ± 0.58	NS
Alb (g/dL)	Before IFN	4.18 ± 0.35	4.10 ± 0.34	4.26 ± 0.36	NS
	2 weeks after IFN	3.76 ± 0.48	3.75 ± 0.38	3.76 ± 0.59	NS
	3 months after IFN	3.84 ± 0.37	3.76 ± 0.45	3.92 ± 0.28	NS
	6 months after IFN	3.88 ± 0.33	3.74 ± 0.42	4.02 ± 0.12	**0**.**0550**
	End of treatment	3.90 ± 0.38	3.77 ± 0.35	4.03 ± 0.39	NS
	6 months after treatment	4.27 ± 0.35	4.23 ± 0.34	4.30 ± 0.39	NS
Salivary flow (g/2 min)	Before IFN	6.10 ± 1.81	5.86 ± 1.90	6.38 ± 1.81	NS
	2 weeks after IFN	5.71 ± 2.60	5.88 ± 2.33	5.55 ± 3.03	NS
	3 months after IFN	5.23 ± 2.14	4.92 ± 2.34	5.53 ± 2.06	NS
	6 months after IFN	5.42 ± 1.97	5.32 ± 2.14	5.51 ± 1.94	NS
	End of treatment	5.31 ± 2.06	5.33 ± 1.58	5.30 ± 2.58	NS
	6 months end of IFN	6.02 ± 1.81	6.03 ± 1.38	6.00 ± 2.28	NS
Diabetes mellitus	positive, n (%)	2 (14.29%)	1 (14.29%)	1 (14.29%)	NS
Oral mucosal lesions	positive, n (%)	7 (50.00%)	6 (85.71%)	1 (14.29%)	**0.0075**
Steroid for external use	positive, n (%)	6 (42.86%)	5 (71.43%)	1 (14.29%)	**0**.**0308**
Tooth-brushing after every meal	yes, n (%)	8 (57.14%)	3 (42.86%)	5 (71.43%)	NS
Denture wearer	yes, n (%)	0 (0%)	0 (0%)	0 (0%)	NS
Administration of antifungal agent	yes, n (%)	2 (14.29%)	2 (28.57%)	0 (0%)	NS
Effect of IFN therapy	SVR , n (%)	11 (78.57%)	6 (85.71%)	5 (71.43%)	NS

HCV, hepatitis C virus; IFN, interferon; M, male; F, female; ND, not detected; NS, not significant; PLT, platelets; Hb, hemoglobin; Alb, albumin.

Body weight decreased significantly in group 1 during IFN therapy, compared to group 2 (P=0.0088). Number of neutrophils, PLT, Alb, and Hb decreased in all subjects throughout the course of IFN treatment. In group 1, the level of Alb at the beginning of the 6^th^ month of IFN administration was lower than in group 2 (P=0.0550, Table 2). The number of times teeth were brushed after every meal was lower in group 1 than group 2.

### Salivary flow

Salivary flow decreased in all subjects throughout the course of IFN treatment and returned at 6^th^ months after the end of treatment (Table 2). Salivary flow was 6.10 ± 1.81 g/2 min before IFN therapy, 5.71 ± 2.60 g/2 min at the beginning of the 2^nd^ week of IFN administration, 5.23 ± 2.14 g/2 min at the beginning of the 3^rd^ month of IFN administration, 5.42 ± 1.97 g/2 min at the beginning of the 6^th^ month of IFN administration, 5.31 ± 2.06 g/2 min at the end of IFN therapy and 6.02 ± 1.81 g/2 min at 6^th^ months after the end of treatment. There was no significant difference in the salivary flow between group 1 and group 2.

### Multivariate analysis

According to multivariate analysis, one factor, the presence of oral mucosal lesions, was associated with the detection of *Candida*. The adjusted odds ratio for the factor was 36.00 (95% confidence interval 2.68-1485.94).

## Discussion

Oral *Candida albicans* is known to have been isolated from patients with OLP [24,25]. Some *Candida albicans* isolates with special virulence attributes might be co-factors which contribute to the development of OLP, especially erosive OLP [32]. The erosive OLP can cause spontaneous pain during eating and tooth-brushing.

There have been few reports of the detection of oral *Candida* before, during and after IFN treatment for chronic hepatitis C. In this study, *Candida* was detected in 50.0% of patients with HCV-related chronic liver disease and who received IFN treatment. The most significant factor associated with *Candida* was the presence of oral mucosal lesions associated with IFN therapy. 85.71% (6/7) of the oral lesions were treated with topical steroids. We found no correlation between HCV genotype, levels of HCV RNA, IFN type, dose or administration schedule, or effect of IFN therapy with *Candida* detection.

Poor oral health has been reported in HCV-infected patients [33-36]. Coates et al. reported that the experience of dental caries was significantly worse in HCV-infected subjects than in patients in general [33]. Griffin et al. found that patients with rheumatoid arthritis, diabetes or a liver condition were twice as likely to have an urgent need for dental treatment as patients who did not have these diseases and documented a high burden of unmet dental care needs among patients with chronic diseases [36]. The authors showed that HCV was the strongest predictor of patients reporting poor oral health. In our study, there was a high rate of detection of candida species in patients who did not brush their teeth after every meal, compared to those who did so. Oral hygiene should be encouraged in patients during IFN treatment.

In our previous study, dental problems delayed the initiation of IFN therapy for up to 105 days [28]. HCV-infected patients treated with IFN should be managed by intensive oral care because of lower resistance to infection during the therapy. With regard to the painful OLP, it is just conceivable that an oral cavity with HCV infection is likely to become less healthy than one without HCV infection. REFRECARE-H® (EN Otsuka Pharmaceutical Co. Ltd. Iwate, Japan) is an oral care gel containing hinokitiol, which can remove stains on the teeth and general oral debris, and is effective in the prevention of breath odor and gum diseases. Hinokitiol has significant antimicrobial efficacy against *Staphylococcus aureus*, *Propionibacterium acnes*, *coronavirus*, *Trichophyton*, and *Candida albicans*[37]. We reported that this oral care gel improved the subjective symptoms of all patients with OLP [38].

IFN therapy for HCV infection is associated with various side effects, including a greater level of fatigue, loss of appetite, weight loss, sleep problems, and depression. In the present study, weight loss during IFN therapy lead to oral candidiasis. Weight loss is considered to be the important risk factor for oral candidiasis. Back-Brito GN et al. demonstrated that eating disorders can lead to an increased oral *Candida* carriage [39].

Our study showed that hypoalbuminemia during IFN therapy might lead to oral candidiasis. We reported previously a strong association between hypoalbuminemia and mortality in a hyperendemic area (X town) of HCV infection in Japan [40] and that hypoalbuminemia was an independent risk factor for the development of OLP [41].

Several studies have shown an association between HCV and Sjögren's symdrome (SS) [42,43]. In this study, we demonstrated that the salivary flow of all patients with HCV infection decreased during IFN treatment and increased again after treatment. Various investigators have reported a high prevalence of oral *Candida* species in patients with SS, compared to healthy controls [44-46].

Japanese HCV-infected patients tend to be older than those in other countries and their older age favors the onset of HCC. In addition, oral candidiasis is underdiagnosed among the elderly [19]. Therefore, we require careful attention to the oral cavities of patients who receive IFN therapy.

## Conclusion

In conclusion, our data show that IFN therapy led to the development of oral mucosal lesions, leading to oral candidiasis, and inhibited salivary secretion. We should pay more attention to oral candidiasis as well as additional oral mucosal lesions, in patients with weight loss during IFN treatment. Patients with HCV infection emphasize the paramount importance of oral management.

## Abbreviations

HCV: Hepatitis C virus; CH-C: Chronic hepatitis C; LC-C: Liver cirrhosis type C; HCC: Hepatocellular carcinoma; IFN: Interferon; RBV: Ribavirin; SVR: Sustained virological response; PLT: Platelets; Hb: Hemoglobin; ALT: Alanine aminotransferase; AST: Aspartate aminotransferase; LDH: Lactate dehydrogenase; T.Bil: Total bilirubin; Alb: Albumin.

## Competing interests

The authors declare that they have no competing interests.

## Authors' contributions

YN carried out most of the data collection and drafted the manuscript. KH cultured *Candida*. MS contributed to data analysis. All authors read and approved the final manuscript.

## Pre-publication history

The pre-publication history for this paper can be accessed here:

http://www.biomedcentral.com/1471-230X/12/155/prepub
